# Serum-derived small extracellular vesicles as biomarkers for predicting pregnancy and delivery on assisted reproductive technology in patients with endometriosis

**DOI:** 10.3389/fendo.2024.1442684

**Published:** 2025-01-17

**Authors:** Ayako Muraoka, Akira Yokoi, Kosuke Yoshida, Masami Kitagawa, Bayasula, Mayuko Murakami, Natsuki Miyake, Reina Sonehara, Tomoko Nakamura, Satoko Osuka, Hiroaki Kajiyama

**Affiliations:** ^1^ Department of Obstetrics and Gynecology, Nagoya University Graduate School of Medicine, Nagoya, Japan; ^2^ Nagoya University Institute for Advanced Research, Nagoya, Japan; ^3^ Japan Science and Technology Agency (JST), FOREST, Saitama, Japan; ^4^ Bell Research Center for Reproductive Health and Cancer, Nagoya University Graduate School of Medicine, Nagoya, Japan

**Keywords:** extracellular vesicles, microRNA, serum, pregnancy, assisted reproductive technology, endometriosis, non-invasive biomarker

## Abstract

**Introduction:**

Endometriosis can cause of infertility, and evaluation methods for predicting clinical pregnancy outcomes are desired. Extracellular vesicles (EVs) exist in blood and it contains small non-coding RNAs (ncRNAs) that may reflect disease severity. In this study, we investigated small ncRNAs in serum EVs to identify specific biomarkers for predicting clinical pregnancy.

**Methods:**

Serum samples were collected from 48 patients who underwent assisted reproductive technology (ART). EVs were successfully isolated from serum samples and characterized using nanoparticle tracking assays, electron microscopy, and western blotting of EV’s markers. We performed small RNA sequencing and analyzed microRNA (miRNA) profiles in the infertility patients with and without endometriosis to detect pregnancy-predicting biomarkers.

**Results:**

Candidate miRNAs in serum EVs were selected by comparing patients without endometriosis who became pregnant (n = 13) with those who did not (n = 21). A total of 241 miRNAs were detected; however, no trends separated the two groups. Next, EVs from patients with endometriosis were analyzed and divided into pregnant (n = 4) and non-pregnant (n = 10) cases. Among the 224 candidate miRNAs, miRNA profiles of pregnant women with endometriosis were separated from those of non-pregnant women by receiver-operating characteristics (ROC) curve analysis (area under the curve [AUC] > 0.8). In patients with endometriosis, serum EVs may be useful for predicting possible pregnancy before infertility treatment. Finally, we used small RNA sequencing of the tissue to demonstrate that pregnancy-predicting miRNAs in serum EVs were produced from endometriosis lesions. Although no predictors were found from miRNAs in serum EVs without endometriosis, miRNAs in serum EVs of patients with endometriosis could provide novel noninvasive biomarkers to predict pregnancy and have potential clinical applicability in ART.

**Discussion:**

Further studies are required to examine the functional importance of these miRNAs to elucidate the pathological mechanisms of endometriosis and pregnancy.

## 1 Introduction

Infertility has received widespread attention owing to its rapid increase among generations in worldwide. As the number of infertile patients increases, the need for assisted reproductive technology (ART) is also growing (1). The International Committee for Monitoring Assisted Reproductive Technologies annual world report series in 2014 estimated that 2 million cycles of *in vitro* fertilization (IVF) and embryo transfer (ET) were conducted worldwide (2). Although ART has provided tremendous benefits to patients with infertility, some limitations remain, including unsatisfactory success rates and limited treatment options (2). To achieve pregnancy, it is critical that the ovaries function in response to ART and the implantation rate in the endometrium. Currently, the ovarian reserve is determined based on the serum concentration of anti-Müllerian hormone (AMH) (3, 4). However, AMH is only an indication of ovarian reserve and not an estimate of the potential to conceive with ART. Hence, novel predictive biomarkers of pregnancy are required.

Endometriosis is a common gynecological disease affecting 10% of reproductive-aged women, and 30–50% of patients with endometriosis are infertile because the endometriosis impairs ovarian function and oocyte quality (5). Treatment options for endometriosis are currently based on hormonal therapy and surgical resection (6); however, patients do not become pregnant under hormonal therapy, and the ovarian reserve is reduced after surgical resection (7, 8). Whether surgery or ART should be performed first when treating patients with endometriosis-induced infertility remains controversial. Moreover, predicting pregnancy rates in patients with endometriosis before treatment is difficult. For patients with endometriosis, it may be useful to consider how likelihood of pregnancy with ART despite the presence of endometriosis lesions prior to ART treatment. It would be advantageous for patients with endometriosis to determine the likelihood of pregnancy after ART in the presence of endometriosis lesions prior to ART treatment.

Extracellular vesicles (EVs), particularly exosomes, are nano-sized membrane-bound vesicles produced by almost all cells that are detected in a variety of body fluids (9, 10). EVs carry several RNA species, such as mRNA, microRNA (miRNA), piwi-interacting RNA (piRNA), and transfer RNA fragments, in cell-to-cell communications (11–13). Many researchers have examined the functional role of intracellular communication through EVs in several conditions, including endometriosis. Small non-coding RNAs (ncRNAs) in serum EVs have recently been identified as a mechanism of intercellular communication and as a source of biomarkers (14–16). Therefore, small ncRNAs in serum EVs are potential noninvasive predictive biomarkers for several outcomes, such as pregnancy.

There are several researches that have revealed the relationships between biomarkers in EVs and endometriosis (17–21). Specific miRNAs in EVs are revealed to influence the pathogenesis of endometriosis and EVs-dependent signaling can exhibit a profound effect on disease progression (22). Moreover, EV research is expanding into the realm of endometriosis-associated infertility, reflecting the regulation of follicular maturation and epigenetic alterations (23).

This study investigated small ncRNAs in the serum EVs of patients with endometriosis as biomarkers for ART-related possible pregnancy.

## 2 Methods

### 2.1 Study population

Serum samples were collected from females undergoing ART at the Nagoya University Hospital. Informed consent was obtained from each patient prior to ovarian stimulation. Consent to publish clinical information potentially identifying individuals (e.g., age, sex, and clinical history) was obtained. This study was approved by the Ethics Committee of Nagoya University School of Medicine (approval number: 2022-0010). Serum samples of infertile patients were obtained on days 3–5 of the menstrual cycle, prior to the start of infertility treatment. The serum sampling cohort from infertility patients without endometriosis was divided into two groups: the pregnancy group (n = 13), comprising patients for whom infertility treatment resulted in at least one pregnancy and delivery, and the non-pregnancy group (n = 21), comprising patients for whom no pregnancy was achieved during ART. Patients with polycystic ovarian syndrome as obvious cause of infertility was excluded from the study. The serum sampling cohort from infertility patients with endometriosis was divided into two groups: the pregnancy group (n = 4) and non-pregnancy groups (n = 10).

### 2.2 Ovarian stimulation and the IVF/intracytoplasmic sperm injection procedure

Ovarian stimulation involved administering urinary follicle-stimulating hormone (FSH) (uFSH Aska, ASKA Pharmaceutical Co., Ltd., Tokyo, Japan) or recombinant FSH (Gonalef, Merck BioPharma, Tokyo, Japan) at 150–300 IU per day for the first two days, after which the doses were adjusted individually based on the follicular response under gonadotropin-releasing hormone antagonist or agonist protocols. Pituitary suppression was achieved by daily administration of ganirelix acetate (GANIREST, Organon & Co., Jersey City, NJ, USA). When the mean follicles diameters reached ≥18 mm, 10,000 IU of human chorionic gonadotropin (HCG for injection, Fuji Pharmaceutical Co. Inc., Toyama, Japan) was administered, and transvaginal oocyte retrieval was performed 35.5 h later. The protocols used for oocyte retrieval and preparation, sperm preparation, and IVF/ICSI have been previously described (24). ICSI or conventional methods were performed, depending on the sperm concentration and motility.

### 2.3 Embryo transfer

In the ET protocol, all patients were treated using the same hormonal replacement therapy (HRT) protocol. A transdermal estradiol patch (Estrana^®^ tape, 0.72 mg; Hisamitsu Pharmaceutical Co. Inc. Tokyo, Japan) was used to stimulate endometrial growth, and Utrogestan (UTROGESTAN vaginal capsules 200 mg; Fuji Pharmaceutical Co. Inc. Tokyo, Japan; or LUTINUS vaginal tablet 100 mg; Ferring Pharmaceuticals Co., Ltd.) was used for luteal phase support.

### 2.4 Sample collection

Serum was collected on days 3–5 of the menstrual cycle, prior to the start of infertility treatment. All serum samples were individually placed into 15-mL conical tubes and centrifuged at 430 ×g for 10 min. The clear supernatant was aliquoted into 2-mL tubes and stored at −80°C until further analysis. Tissue samples were obtained from 10 different patients, who had regular menstrual periods and did not receive any hormonal treatment for at least 3 months before operation. Each small piece of tissue was quick frozen and stored at −80°C until further analysis.

### 2.5 EV isolation

Approximately 1 mL of each serum sample was centrifuged at 10,000 ×g for 40 min at 4°C in a Kubota Model 7000 ultracentrifuge. The supernatant was filtered using a 0.22 µm filter (Millex-GV 33 mm, Millipore), and then ultracentrifuged at 110,000 ×g for 70 min at 4°C using an MLS50 rotor (Beckman Coulter Inc., USA). The pellet was washed with phosphate buffered saline (PBS), ultracentrifuged under the same conditions, and resuspended in PBS to extract the small EVs. The protein concentrations of the EVs and cell lysates were quantified using a Qubit protein assay kit (Thermo Fisher Scientific) with a Qubit 4.0 Fluorometer (Invitrogen Co., MA, USA), according to the manufacturer’s protocol. The size distribution and particle concentration of the EV preparations were analyzed using a NanoSight NS300 nanoparticle tracking analyzer (Malvern Panalytical Ltd., UK). The samples were diluted in PBS and injected at a speed of 100 a.u. into the measuring chamber. The EVs flow was recorded in triplicate (30 s each) at room temperature. The equipment settings for data acquisition were maintained constant between measurements, with the camera level set to 13.

### 2.6 Transmission electron microscopy

After post-fixing in 2% osmium tetroxide for 3 h at room temperature, the samples were dehydrated using ascending ethanol concentrations (50–100%). The samples were stained with aqueous uranyl acetate and examined under a transmission electron microscope (LEM-1400PLUS, JEOL Ltd.).

### 2.7 Western blotting analysis of EVs and cell lysates

Samples of serum EVs prepared with adjusted amounts of protein were loaded onto polyacrylamide gels for the electrophoretic separation of proteins at 20 mA. After blocking with skim milk (Snow Brand Megmilk Co., Japan) or Blocking One (Nacalai Tesque Inc., Japan) for 1 h at room temperature, the membranes were incubated overnight at 4°C with the following primary antibodies: mouse monoclonal anti-CD9 (CBL162, Merck; 1:100), rabbit monoclonal anti-CD63 (EXOAB-CD63A-1, System Biosciences, LLC, CA, USA; 1:1,000), and mouse monoclonal anti-CD81 (sc-166029, Santa Cruz Biotechnology, TX, USA; 1:100). The membranes were subsequently washed three times for 5 min each using Tris-buffered saline with 0.1% Tween^®^ 20 (TBST) and incubated for 1–3 h at room temperature with secondary HRP-conjugated mouse anti-rabbit IgG (NA934-1ML, Cytiva Lifesciences, USA; 1:5,000) or anti-mouse IgG (NA931-1ML, Cytiva; 1:2,000) antibodies. The MagicMark™ XP Western Protein Standard (Thermo Fisher Scientific) was used. The membranes were imaged using an ImageQuant LAS 4010 (GE Healthcare, IL, USA). The uncropped blots are shown in **Supplementary Figure S1**.

### 2.8 Small RNA sequencing

RNA was extracted from EVs of serum and tissue samples by using the miRNeasy Plus Mini Kit (QIAGEN, Hilden, Germany). The total RNA concentration of each sample was measured using the Qubit RNA HS Assay Kit (Thermo Fisher Scientific, Waltham, MA, USA). Small RNA libraries were prepared using the NEBNext Multiplex Small RNA Library Prep Set for Illumina (New England Biolabs, Ipswich, MA, USA), and index codes were added to attribute the sequences to each sample. Next, the PCR products were purified using a QIAquick PCR Purification Kit (Qiagen) and a 6% TBE gel (120 V, 60 min). Furthermore, DNA fragments corresponding to 140–160 bp (the length of the small ncRNA plus the 3′ and 5′ adaptors) were recovered, and the complementary DNA concentrations were measured using the Qubit dsDNA HS Assay Kit and a Qubit2.0 Fluorometer (Life Technologies, Carlsbad, CA). Single-end reads were obtained using an Illumina MiSeq or NextSeq (Illumina, San Diego, CA, USA).

### 2.9 Bioinformatics analysis

The small RNA sequencing raw data files were analyzed with the CLC Genomics Workbench version 9.5.3 program (Qiagen). After adaptor trimming, the data were mapped to the miRbase 22 database, allowing up to two mismatches, and normalized using reads per million (RPM) mapped reads. RStudio (RStudio, Boston, MA, USA) and R (ver. 4.0.3) were used. To visualize the volcano plots, the log2 fold change (log2 FC) and adjusted p-values for each gene were calculated using the Wald test in DESeq2 (ver. 1.30.0). After excluding miRNAs with a maximum RPM of <100, 241 miRNAs were selected for subsequent analysis of serum EVs from infertility patients without endometriosis. 224 miRNAs in serum EVs that were differentially expressed between pregnant and non-pregnant patients with endometriosis were selected using the *t*-test with an adjusted p-value <0.05 and log2 FC >0.7 as the cutoff criteria.

## 3 Results

### 3.1 Characterization of serum EVs and miRNA isolation

**Figure 1:**
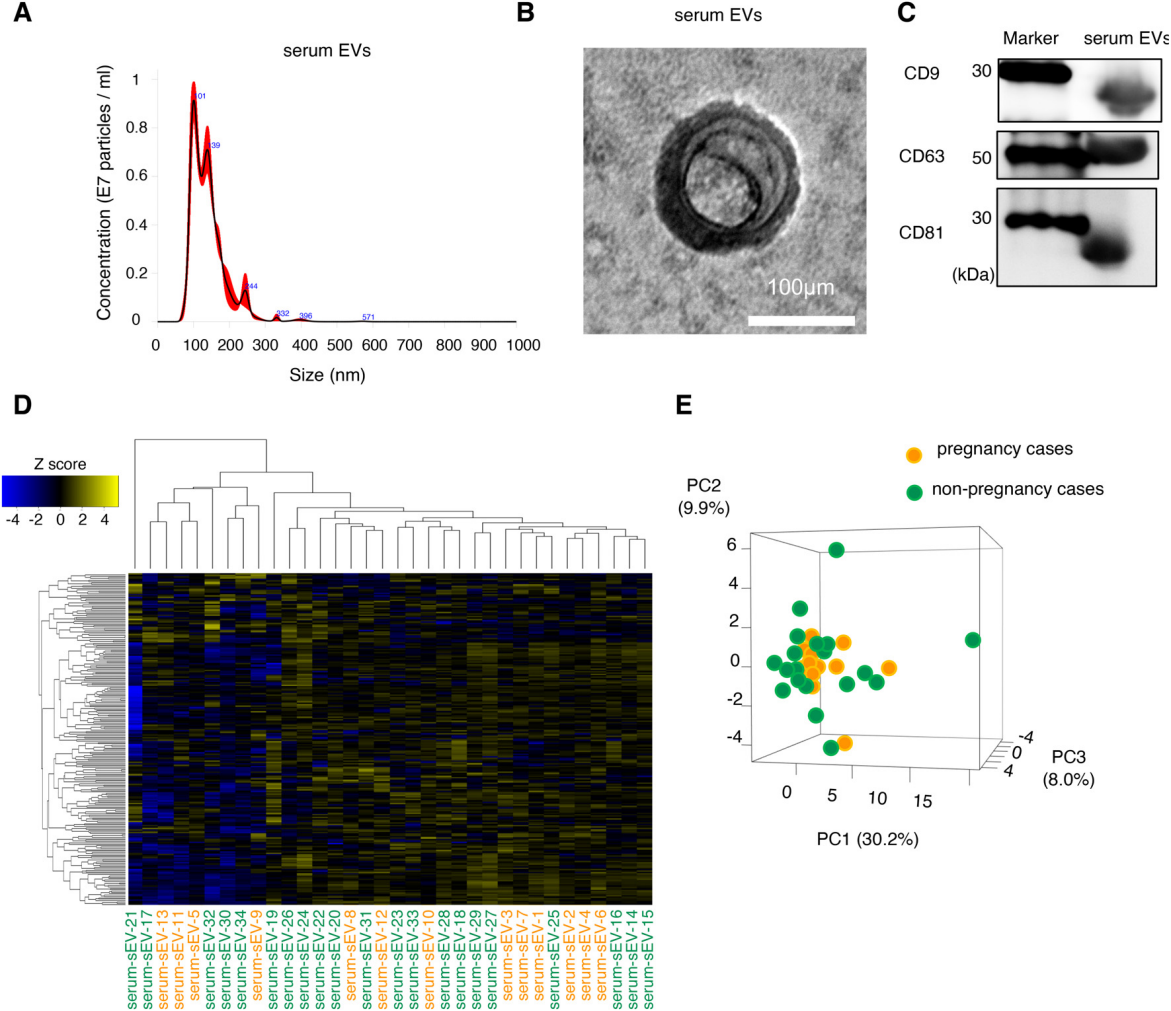
Characterization and expression analysis of serum EVs. **(A)** Size distribution of EVs determined by a nanoparticle tracking analysis. **(B)** Morphology of the EVs detected by transmission electron microscopy. The scale bar represents 100 nm. **(C)** The protein expression of EV markers (CD9, CD63, and CD81). **(D)** The clustering and heatmap analyses of the miRNA profiling in serum EVs from patients without infertility cause. Orange and green letters indicate pregnancy cases (serum-EVs-1 to -13) and non-pregnancy cases (serum-EVs-14 to -34), respectively. The normalized data were converted to base 10 logarithms and z-scores. **(E)** Principal component analysis (PCA) of the miRNA profiling is shown in **(D)**. PC, principal component.

We selected the pregnant patients (patients without endometriosis; n = 13; patients with endometriosis, n = 4) and non-pregnant patients (patients without endometriosis, n = 21; patients with endometriosis, n = 10) for analysis. EVs were isolated from each serum sample. The serum EVs had an average diameter of 130 nm, as determined by nanoparticle tracking analysis, and included small EVs because their diameter was ≤200 nm (**Figure 1A**). The morphology of the isolated EVs was confirmed by electron microscopy (**Figure 1B**). Serum EVs were positive for CD9, CD63, and CD81, which are well-known major exosomal markers (**Figure 1C**). Based on this data, we had isolated the EVs from serum samples.

### 3.2 Expression analysis of miRNAs in serum EVs from patients without endometriosis

First, we examined serum EVs from patients without endometriosis to detect candidate miRNAs to predict the possible pregnancy before the initiation of infertility treatment. Serum samples from pregnant (n = 13) and non-pregnant (n = 21) were analyzed using small RNA sequencing, and their characteristics are shown in **Table 1**. The average age in the pregnancy group was significantly lower than that in the non-pregnancy group; however, the causes of infertility, duration of infertility, ART method, and body mass index (BMI) were not significantly different between the two groups. Small RNA sequencing identified 241 candidate miRNAs, and hierarchical clustering and heatmap analyses revealed the miRNA profiles (**Figure 1D**, **Supplementary Table S1**). In principal component analysis (PCA), miRNA profiles were not clearly divided between the two groups; thus, miRNA profiles in serum EVs from patients without endometriosis were not directly associated with pregnancy outcomes (**Figure 1E**).

**Table 1:** Characteristics of non-endometriosis group.

Variable		Pregnant cases (n = 13)	Non-pregnant cases (n = 21)	p-value
Age, years (± SD)		33 ± 4.1	36 ± 3.6	0.04 *^a^*
Diagnosis	Primary infertility	9	15	1b
	Secondary infertility	4	6	
Duration of infertility, years	<5	11	19	0.54^b^
	6–10	2	1	
	>10	0	1	
ART method	IVF	2	3	1^b^
	ICSI	11	19	
BMI, kg/m^2^ (± SD)		21 ± 3.7	21 ± 3.6	0.56 *^a^*

BMI, body mass index; ART, assisted reproductive technology; IVF, *in vitro* fertilization; ICSI, intracytoplasmic sperm injection; SD, standard deviation.

a Student t-test.

b Fisher’s exact test.

### 3.3 Expression analysis of miRNAs in serum EVs from patients with endometriosis

**Figure 2:**
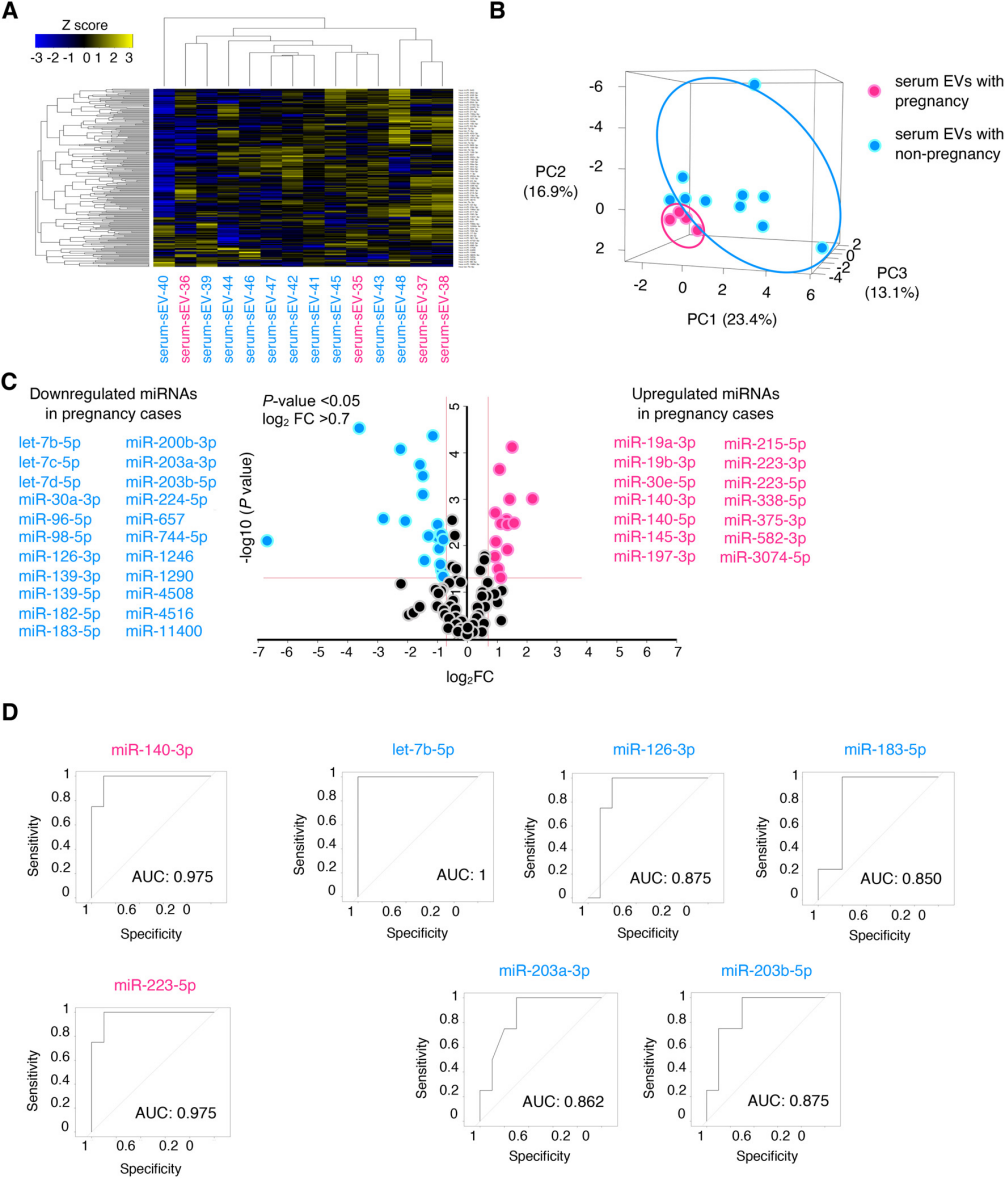
Expression analysis of miRNAs in serum EVs of patients with endometriosis. **(A, B)** The heatmap and PCA of the miRNA profiling in serum EVs of patients with endometriosis. Magenta and turquoise letters indicate pregnancy (serum-EVs-35 to -38) and non-pregnancy cases (serum-EVs-39 to -48), respectively. PC, principal component. The normalized data were converted to base 10 logarithms and z-scores. **(C)** Volcano plot of dysregulated genes generated by the miRNA profiling shown in **(A)** Upregulated and downregulated miRNAs in pregnancy cases are listed. **(D)** ROC curves for detecting pregnancy-predicting biomarkers using each of the seven miRNAs. The AUC was calculated, and >0.8 was selected.

Next, we speculated whether serum EVs could be predictive biomarkers of pregnancy outcomes among patients with infertility diseases, such as endometriosis. We selected another patient cohort with endometriosis; serum samples from pregnant (n = 4) and non-pregnant patients (n = 10) were analyzed by small RNA sequencing, and their characteristics are shown in **Table 2**. The age, cause of infertility, duration of infertility, ART method, BMI, and endometriosis cyst diameter were not significantly different between the two groups. The severity of endometriosis is typically assessed using the revised American Society for Reproductive Medicine (r-ASRM) score; however, as many patients in the cohorts did not obtain their r-ASRM score because they underwent operations in other hospitals, the endometriosis cyst size was used to compare the two groups. Small RNA sequencing identified 224 miRNAs that were candidates, and hierarchical clustering and heatmap analyses revealed the miRNA profiles (**Figure 2A**, **Supplementary Table S2**). In PCA, the miRNA profiles were clearly divided between the two groups (**Figure 2B**). Among pregnant women, 14 miRNAs were significantly upregulated in serum EVs from patients with endometriosis, whereas 22 miRNAs were significantly downregulated compared to miRNAs in serum EVs from non-pregnant patients with endometriosis of non-pregnant women (**Figure 2C**). Receiver operating characteristics (ROC) curve analysis and the area under the curve (AUC) were used to validate 36 miRNAs. The seven ROC curves with an AUC >0.8 and a normalized read count of more than a thousand are shown in **Figure 2D**.

**Table 2:** Characteristics of endometriosis group.

Variable		Pregnant cases (n = 4)	Non-pregnant cases (n = 10)	p-value
Age, years (± SD)		35 ± 1.5	36 ± 4.3	0.48 *^a^*
Diagnosis	Primary infertility	4	9	1^b^
	Secondary infertility	0	1	
Duration of infertility, years	<5	4	8	0.055^b^
	6–10	0	1	
	>10	0	1	
ART method	IVF	1	2	1^b^
	ICSI	3	8	
BMI, kg/m^2^ (± SD)		20 ± 2.9	21 ± 2.1	0.73 *^a^*
Cyst size, cm (± SD)		5 ± 1.4	6 ± 2.2	0.47 *^a^*

BMI, body mass index; ART, assisted reproductive technology; IVF, *in vitro* fertilization; ICSI, intracytoplasmic sperm injection; SD, standard deviation.

a Student t-test.

b Fisher’s exact test.

### 3.4 Verification of whether miRNAs are produced by endometriosis lesions

**Figure 3:**
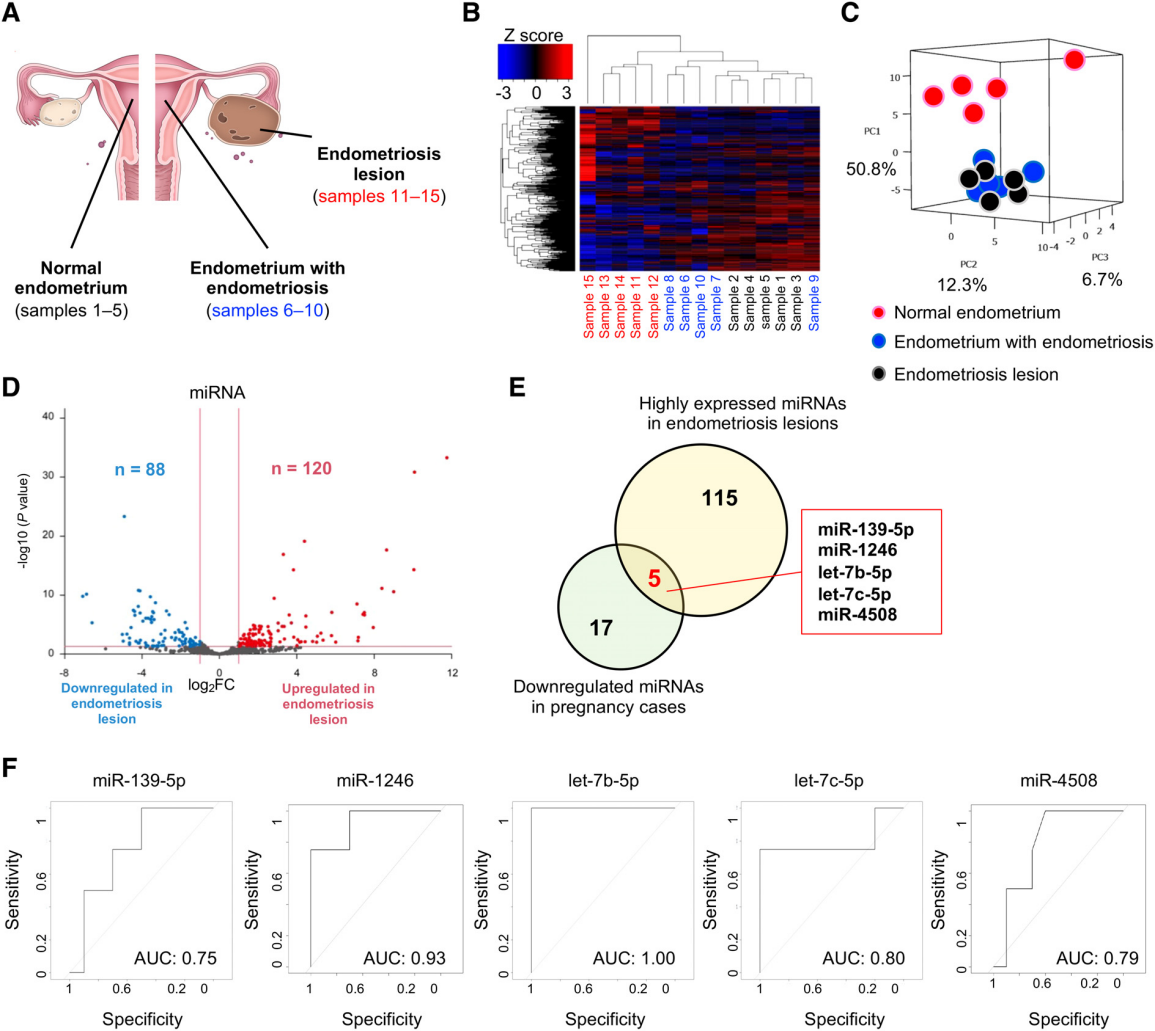
Expression analysis of miRNAs in tissue samples. **(A)** Schema of the samples. **(B, C)** The heatmap and PCA of the miRNA profiling in tissue samples. Black, blue, and red letters indicate normal endometrium (samples 1–5)), endometrium with endometriosis (samples 6–10), and endometriosis lesions (samples 11–15), respectively. PC, principal component. The normalized data were converted to base 10 logarithms and z-scores. **(D)** Volcano plot of dysregulated genes in endometriosis lesions generated by the miRNA profiling shown in **(B)**. **(E)** The Venn diagram shows the five specific miRNAs (miR-139-5p, miR-1246, let-7b-5p, let-7c-5p, and miR-4508). Yellow and green circles indicate the highly expressed miRNAs in endometriosis lesions and downregulated miRNAs in pregnancy cases, respectively. **(F)** ROC curves for detecting pregnancy-predicting biomarkers in serum samples using each of the five miRNAs.

To detect the original organs that produce pregnancy-predicting miRNAs in serum EVs, we extracted small RNAs from tissue samples of the normal endometrium (sample nos. 1–5), endometrium with endometriosis (sample nos. 6–10), and endometriosis lesions (sample nos. 11–15) (**Figure 3A**). Patient characteristics are shown in **Table 3**. Small RNA sequencing identified 547 miRNAs for analysis, and hierarchical clustering and heatmap analyses revealed clearly divided miRNA profiles (**Figure 3B**). In PCA, the miRNA profiles were clearly divided between the normal endometrium (samples 1–5) and endometriosis groups (samples 6–15) (**Figure 3C**). In endometriosis lesions, 120 miRNAs were significantly upregulated, whereas 88 miRNAs were significantly downregulated compared to normal endometrium (**Figure 3D**). Of the 120 miRNAs highly expressed in endometriosis lesions, 5 miRNAs were detected in the pregnancy-predicting miRNAs, which were downregulated in the pregnancy group (miR-139-5p, miR-1246, let-7b-5p, let-7c-5p, and miR-4508) (**Figure 3E**). Overall, 5 miRNAs were validated using ROC analysis and an AUC >0.75 (**Figure 3F**). Based on these data, some potential pregnancy-predicting miRNAs markers in serum EVs were produced by endometriosis lesions.

**Table 3:** Patient characteristics of tissue samples.

Variable	Non-endometriosis (n = 5)	Endometriosis (n = 5)	p-value
Age, years (± SD)	32 ± 5.4	39 ± 3.8	0.052 *^a^*
BMI, kg/m^2^ (± SD)	20 ± 1.4	20 ± 1.1	0.57 *^a^*
Cyst size, cm (± SD)	N.A.	7 ± 3.2	

BMI, body mass index; SD, standard deviation; N.A., not applicable.

a Student t-test.

## 4 Discussion

If a biomarker can be measured before a certain treatment is administered and the effect of that treatment can be predicted, it can be a criterion by which a patient chooses a treatment. In reproductive medicine, predicting the possible pregnancy prior to ART would make it possible to predict post-treatment outcomes at the desired time of conception for each individual. In this study, we identified differentially expressed miRNAs in serum EVs of patients with endometriosis to predict pregnancy after ART. While the analysis of serum EVs of patients without endometriosis revealed no particular biomarkers, certain miRNAs that could predict possible pregnancy were detected in serum EVs of patients with endometriosis. Furthermore, pregnancy-predicting miRNAs in serum EVs originated from endometriosis lesions, suggesting that endometriosis is associated with infertility.

miRNAs play important roles in a wide range of biological processes, such as development, cell proliferation, differentiation, apoptosis, and metabolism, in diverse animals, and miRNAs in serum EVs show specific expression patterns related to several diseases and may reflect the other organ functions. First, we examined the miRNAs in the serum EVs of infertile patients without endometriosis to predict possible pregnancy before starting infertility treatment. However, no candidate biomarkers predicting pregnancy were identified in our analysis. The reasons for this include the possibility that a small proportion of miRNAs in serum EVs are expressed as indicators of ovarian function, and individuals may have large differences. Therefore, we focused on endometriosis as an infertility-related disease and examined the miRNAs in serum EVs from different patient cohorts. Endometriosis is a common gynecological disease, and 30–50% of patients are infertile due to ovarian damage and pelvic inflammation (25). In the previous studies, several miRNAs in serum samples have been detected as diagnostic markers for endometriosis, some of which may be related to the endometriosis severity and pathological functions. Of the differentially expressed miRNAs in serum EVs in our study, miR-139-3p (26) and 200b-3p (27) were downregulated in pregnant women, and these miRNAs have been reported to be differentially expressed in serum samples of endometriosis patients compared to control patients. Several candidate miRNAs detected in our study have been reported as endometriosis-associated miRNAs in previous studies. For example, miR-126 enhances vascular endothelial growth factor (VEGF) and fibroblast growth factor (FGF) signaling via the suppression of inhibitors of these pathways, leading to neo angiogenesis and the development of mature vasculature in endometriosis (27). In the present study, the expression of miR-126 was downregulated in pregnant women. This may mean that miR-126-mediated neo angiogenesis-promoting effect is suppressed in endometriosis. It suggests that the pathogenesis of endometriosis may be milder and associated with better pregnancy outcomes. In our study, miR-223-3p and -5p were upregulated in pregnant women. miR-223, which is not downregulated in pregnant patients with endometriosis in our study, is the most abundant miRNA in EVs derived from macrophages and can be captured by epithelial, endothelial, and fibroblast cells in endometriosis lesions (28). This miRNA plays an important role in increasing the activity of M2 macrophages, which affects the anti-inflammatory status (28, 29). The high expression of miR-223 in pregnant women may reduce the severity of endometriosis via activating M2 macrophages and lead to better pregnancy outcomes. However, these notions are based on the hypothesis that miRNAs functioning in endometriosis lesions are secreted from tissues into the bloodstream; therefore, further functional analysis is required. Finally, to detect the origin of pregnancy-predicting miRNAs in serum EVs, we analyzed small RNA sequences from tissue samples of the normal endometrium, endometrium with endometriosis, and endometriosis lesions. In our analysis, five types of miRNAs were highly expressed in endometriosis lesions and could predict pregnancy in patients with endometriosis. One candidate, miR-139-5p, is significantly upregulated in endometriosis lesions (30–32) and may play a key role in the progression of endometriosis by regulating the viability of endometrial stromal cells and directly targeting Bcl-2-binding component 3 (31). Detecting miRNAs involved in the pathogenesis of endometriosis in the serum suggests that they are likely to be informative biomarkers.

This study had several limitations. First, we could only select a relatively small number of pregnancy cases because of low pregnancy rates. Further large-scale studies are required, including samples from multiple institutions. Second, although some of the miRNAs identified in this study provided a convincing explanation for the differentially expressed miRNAs in pregnancy, others exhibited the opposite direction of dysregulation in different studies. Differential endpoints, miRNA containers, technical differences in sample handling, RNA extraction, normalization, and statistical analytical methods are likely to account for some of the differences between studies. Further studies are required to confirm these findings. Finally, the functional analysis of candidate miRNAs and detection of target genes should be validated in future studies to reveal their functions.

In conclusion, this is the first report to identify miRNA biomarkers in EVs for predicting pregnancy using comprehensive, highly sensitive small RNA sequencing. Certain miRNAs in the serum EVs of patients with endometriosis could predict possible pregnancy before starting the infertility treatments. Our findings could contribute to novel noninvasive pregnancy predictors that are clinically applicable to patients undergoing ART.

## Data Availability

The datasets presented in this study can be found in online repositories. The names of the repository/repositories and accession number(s) can be found below: https://www.ncbi.nlm.nih.gov/, GSE266260, https://www.ncbi.nlm.nih.gov/, GSE266261.

## Supplementary material

Supplementary Figure 1 Uncropped blots. **(A)** Uncropped blots for western blotting.

Supplementary Table 1 List of 241 candidates miRNAs.

Supplementary Table 2 List of 224 candidates miRNAs.
