# Supplementary figures and images for: Serum-derived small extracellular vesicles as biomarkers for predicting pregnancy and delivery on assisted reproductive technology in patients with endometriosis
Source: Front Endocrinol (Lausanne). 2025 Jan 17;15:1442684. doi: 10.3389/fendo.2024.1442684 (PMC11782015; doi:10.3389/fendo.2024.1442684)

**A**

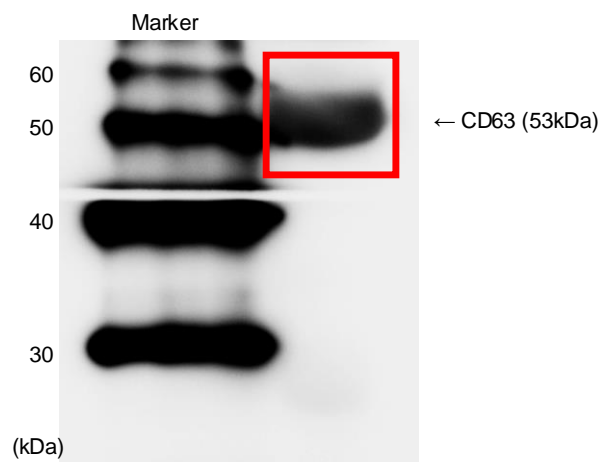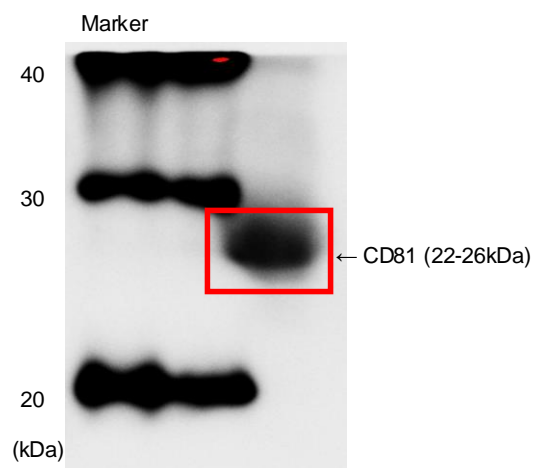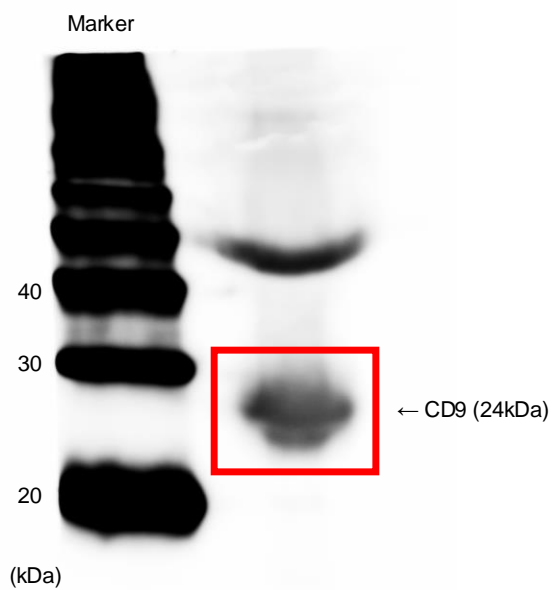

Supplement: Supplementary Figure 1 — Uncropped blots. (A) Uncropped blots for western blotting. [file Image1.pdf]
